# Ionic Liquids and Cellulose: Dissolution, Chemical Modification and Preparation of New Cellulosic Materials

**DOI:** 10.3390/ijms150711922

**Published:** 2014-07-04

**Authors:** Mehmet Isik, Haritz Sardon, David Mecerreyes

**Affiliations:** 1 POLYMAT, University of the Basque Country UPV/EHU, Avda. Tolosa 72, 20018 San Sebastian, Spain; E-Mails: isik.mehmet@ehu.es (M.I.); haritz.sardon@ehu.es (H.S.); 2 Ikerbasque, Basque Foundation for Science, E-48011 Bilbao, Spain

**Keywords:** cellulose, ionic liquid, polymerized ionic liquid, composite

## Abstract

Due to its abundance and a wide range of beneficial physical and chemical properties, cellulose has become very popular in order to produce materials for various applications. This review summarizes the recent advances in the development of new cellulose materials and technologies using ionic liquids. Dissolution of cellulose in ionic liquids has been used to develop new processing technologies, cellulose functionalization methods and new cellulose materials including blends, composites, fibers and ion gels.

## 1. Introduction

**Figure 1:**
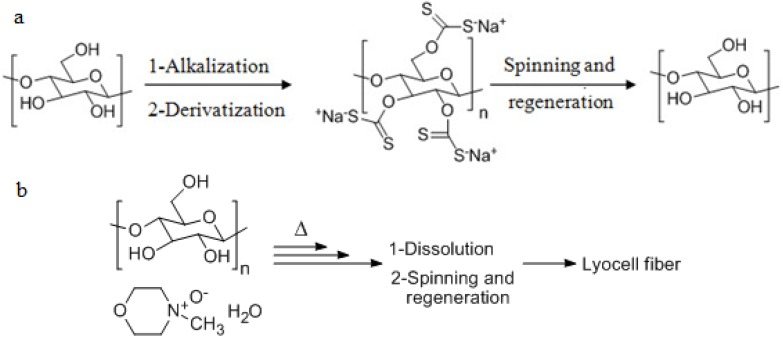
Chemical representation of two industrially important cellulose technologies (**a**) Viscose and (**b**) Lyocell processes. Δ = heat provided to the system.

Cellulose is the most abundant natural polysaccharide on earth being the main structural component of plant cell walls and some seaweed [1,2,3,4]. Cellulose is formed from repetitive d-glucose units, which are linked through β(1→4)-glycosidic bonds [5]. This natural polysaccharide has become one of the most used biomaterials due to its fascinating structural and physical properties and biocompatibility. These properties arise from the multiple hydrogen bonding interactions resulting in a semicrystalline polymer containing highly structured crystalline regions, which form materials with high tensile strength. Although Anselme Paven discovered cellulose in 1838, the first cellulose-based thermoplastic material was produced in 1870 by the Hyatt Manufacturing Company. This material was manufactured by treating cellulose with nitric acid to form cellulose nitrate and commercialized under the trade name “celluloid” [6,7]. A couple of years later, viscose, a new process to regenerate cellulose fibers in a larger scale, was developed. This process rendered possible the utilization of cellulose in different fields such as textile industry, construction, ceramics, paints, cosmetics or food industry [8,9]. A newer technology, in comparison to viscose production, the Lyocell process, was introduced into the market during the 1980s and uses direct dissolution of cellulose to produce lyocell fibers mainly for the textile industry [10]. The major problem associated with this process is that the amine oxide solvent suffers from the drawback that the regeneration involves dangerous and potentially explosive conditions [11]. A general scheme for the processing of cellulose for these two industrially important processes is given below in Figure 1. 

Cellulose is obtained mainly from four resources; forestry, agricultural crops, industrial and animal residues. The extracted biomass that is obtained from all those sources contains three major components: cellulose, lignin and hemicellulose with percentages ranging from 40% to 50%, 18% to 35%, and 25% to 35%, respectively. The percentages of the components strongly depend on the employed source [12]. Thus, the extracted biomass has to be processed in order to separate the different components and isolate the cellulose. There are three major industrially employed processing or pulping technologies called sulfite, organosolv and Kraft processes [13]. Although the Kraft process is the most widely used pulping method, there are fatal drawbacks related to the use of this process such as the degradation of lignin and hemicellulose, the utilization of high temperature and pH, the release of organic sulfur compounds or the water contamination. On account of these, the major challenge is the separation and purification of the biomass without destroying the lignin and hemicellulose using more benign strategies that do not include the use of toxic and non-recyclable chemicals [14]. 

**Figure 2:**
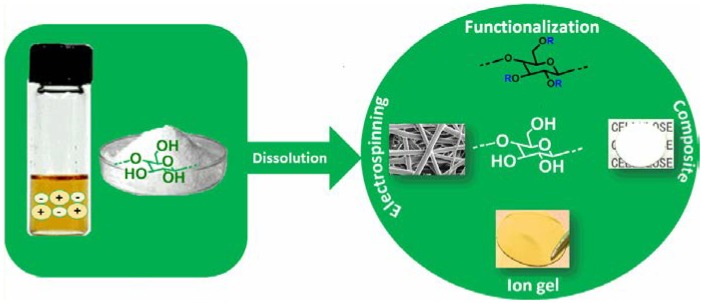
Possible materials and technologies generated from cellulose dissolved in ionic liquids.

Due to the drawbacks associated with the current methodologies used to dissolve and process cellulose [15], environmentally friendly and more efficient solvents are required. In the last decade, ionic liquids have emerged as effective and green solvents, mainly due to their high thermal and chemical stability, nonflammable nature and miscibility with many other solvent systems. In the early 2000s Swatloski *et al.* [16] discovered the ability of some ionic liquids to dissolve cellulose, which afterwards provoked a high interest in this area. This review summarizes the most notable ionic liquid and cellulose materials and technologies developed after this discovery, focusing on the dissolution of cellulose, its chemical modification and the processing and preparation of cellulose composites as designated in Figure 2.

## 2. Ionic Liquids and Cellulose Dissolution

### 2.1. Cellulose Dissolution in Ionic Liquids

The first report on cellulose dissolution in ionic liquids was published in 2002 by Rogers *et al.* [16]. To give an idea of the impact of this discovery, nowadays in 2014 this pioneer article has received more than 2000 scientific citations being one of the most cited articles of the year by the prestigious Journal of the American Chemical Society. In this study, ionic liquids combining 1-butyl-3-methyl imidazolium cation with different anions were investigated as solvents of cellulose. It was found out that chloride, as a small hydrogen bond acceptor, was the most effective anion to dissolve cellulose in comparison to large, non-coordinating anions. Since then, many ionic liquids have been reported in the literature with the ability to efficiently dissolve cellulose, such as the ones with halide counter ions like 1-butyl-3-methyl-imidazolium chloride (BMIMCl) and other counter anions such as phosphate, formate and acetate [16,17]. One disadvantage associated with the ionic liquids with halide anions is relatively high viscosities which brings processing difficulties during the dissolution process. However ionic liquids with anions such as acetate, formate and phosphate possess lower viscosities that facilitate their use for various applications [17]. Therefore, commercially available cellulose solutions today are prepared with 1-ethyl-3-methylimidazolium acetate ionic liquid due to its lower viscosity and high cellulose dissolving ability. In many studies with ionic liquids, it was foreseen that the anion is of great importance and responsible for the dissolution of cellulose and the role of the cation was not that important. However, some recent studies have shown that not only the structure of the anion is important but also the structure of the cation is relatively significant in the solvation process. Thus, acidic protons on the heterocyclic rings increase substantially the solubility by forming hydrogen bonds with hydroxyl and ether oxygen of cellulose [18]. While 1-butyl-3-methylimidazolium acetate, known nowadays as one of the best ionic liquids, displayed 23 g/mol solubility at 40 °C, changing the cationic structure to 1-methoxyethyl-3-methylimidazolium resulted in a dramatic decrease to 8 g/mol solubility at the same temperature. Nowadays, the maximum values of solubility of cellulose were found to be 14.5 wt % for 1-allyl-3-methylimidazolium chloride at 80 °C [19] and 16 wt % for 1-ethyl-3-methylimidazolium acetate at 90 °C [20], which can be increased up to 25 wt % with microwave heating. For rational design of ionic liquids for efficient cellulose dissolution, anions possessing strong hydrogen bond acceptability, cations including strong acidic protons without having high electronegativity atoms such as oxygen and bulky groups that can create steric hindrance should be given priority. The presence of electronegative atoms on the cation will decrease the acidity of the protons causing a decrease in the solvation efficiency. With the aim of improving the dissolution of cellulose in the ionic liquid, dimethyl sulfoxide, DMSO, was also added as a co-solvent. The addition of an organic co-solvent such as DMSO can be used to enhance the solvent power of the ionic liquid by decreasing the time needed for dissolution, even at low temperatures [21,22]. 

Since the first report showing the power of ionic liquids to dissolve cellulose, many ionic liquids have been investigated either to dissolve or to create an appropriate media for the functionalization of cellulose. Table 1 summarizes the most commonly used ionic liquids.

### 2.2. Dissolution of Different Polysaccharides in Ionic Liquids

It is worth to remark that besides cellulose, ionic liquids have shown the ability to dissolve other polysaccharides. Similarly, ILs have been used as a solvent for many biopolymers that are linked together by strong intermolecular hydrogen bonds such as chitin, chitosan, galactomannan or starch. For instance, chitosan, the *N*-deacetylated product of chitin, is the second most abundant biopolymer. It was reported by Xie *et al.* that 1-butyl-3-methylimidazolium chloride can dissolve up to 10 wt % chitin or chitosan in 5 h [23]. In a similar study, native chitin, which has a more complex inter- and intra-molecular hydrogen bond network than cellulose due to the presence of acetoamide groups in the repeat units, was dissolved in room temperature ionic liquid 1-butyl-3-methylimidazolium acetate by Wu *et al.* [24]. Another polysaccharide that can be dissolved in ionic liquids is galactomannan. Lacroix *et al.* [25] used imidazolium based ionic liquids to dissolve guar gums of high molecular weights, which were then modified in varying ionic liquids for the first time through esterification reactions with acid chlorides. It was shown that the chain integrity of the biopolymer was preserved since mild conditions were applied for the dissolution process. In another study, three different galactomannans with different degrees of branching were processed with imidazolium ionic liquids to obtain composite materials [26].

On the other hand, starch, being the major component in many food plants, can also be dissolved and processed with ionic liquids to give materials with tunable properties. Liu *et al.* [27] utilized 1-ethyl-3-methylimidazolium acetate ionic liquid to dissolve waxy corn starch. The rheological properties of the solutions with varying solid contents were examined. It was discovered that the intrinsic viscosity was much less temperature sensitive than cellulose. Fort *et al.* used 1-butyl-3-methylimidazolium chloride ionic liquid to screen fruit ripening [28]. Banana pulps at any stage of ripening were dissolved in the ionic liquid and the compositions were analyzed by high-resolution ^13^C NMR (nuclear magnetic resonance) spectroscopy. The analysis of ^13^C NMR revealed that the bananas contain mostly starch at the early stages of ripening. As the ripening proceeds starch is gradually converted to sucrose, glucose and fructose through an enzymatic degradation process. 

**Table 1:** Structures of some ionic liquids and the extent of cellulose solubility in these ionic liquids. MCC: microcrystalline cellulose, DP: degree of polymerization.

Ionic Liquid and Its Chemical Structure			Temp. (°C)		Solubility (wt %)		Type of Cellulose		Ref.		
1-allyl-3-methylimidazolium chloride ([Amim][Cl])	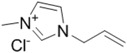		90		5		MCC Avicel		[19]		
			100		10		MCC (DP:250)		[29]		
			100–130		5–14.5		pulp cotton linter		[19]		
1-allyl-2,3-dimethylimidazolium bromide ([ADmim][Br])	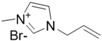		80		12		Avicel		[30]		
			80		4		cotton linters		[30]		
1-allyl-3-methylimidazolium formate ([Amim][HCOO])	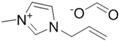		85		22		MCC (DP:250)		[29]		
1-butyl-3-methylimidazolium aminoethanoate ([C_4_mim][H_2_NCH_2_COO])	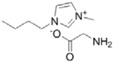		70		12		MCC		[31]		
1-butyl-3-methylimidazolium benzoate ([C_4_mim][PhCO_2_])	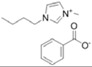		70		12		MCC		[31]		
1-butyl-3-methylimidazolium chloride ([C_4_mim][Cl])	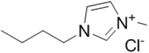		90		<5		MCC Avicel		[20]		
			100		10		dissolving pulp		[16]		
			110		10		MCC Avicel		[32]		
			83		18		MCC Avicel		[33]		
			83		13		suprice sulfite pulp		[33]		
			83		10		cotton linters		[33]		
			100		20		MCC (DP:250)		[29]		
			100		20		MCC Avicel		[34]		
			85		13.6		-		[35]		
1-butyl-3-methylimidazolium formate ([C_4_mim][HCOO])	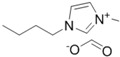		110		8		MCC Avicel		[32]		
1-butyl-3-methylimidazolium dicyanamide ([Bmim][N(CN)_2_])	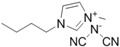		110		1		MCC Avicel		[32]		
1-butyl-3-methylimidazolium bis[(trifluoromethyl)sulphonyl]imide ([Bmim][TFSI])	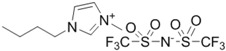		110		0.5		MCC Avicel		[32]		
1-ethyl-3-methylimidazolium chloride ([Emim][Cl])	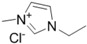		90		5		MCC Avicel		[20]		
1-ethyl-3-methylimidazolium acetate ([Emim][Ac])	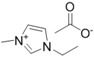		90		16		MCC Avicel		[20]		
			85		13.5		Eucalyptus pulp		[35]		
			110		15		MCC Avicel		[32]		
1-ethyl-3-methylimidazolium diethylphosphate ([Emim][DEtPO_4_])	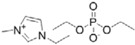		100		12–14		Avicel		[34]		
1-ethyl-3-methylimidazolium methylphosphonate [Emim][(MeO)(H)PO_2_]	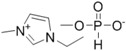		45		10		MCC (DP:250)		[36]		
			25		4		MCC (DP:250)		[36]		
*N,N*-dimethyl-2-methoxyethylammonium acetate ([MM(MeOEt)NH][OAc])	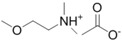		110		12		MCC Avicel		[32]		
*N*,*N*-dimethylethanolammonium acetate ([MM(EtOH)NH][OAc])	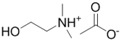		110		<0.5		Avicel		[32]		
3-methyl- *N*-butylpyridinium chloride ([MNBuPy][Cl])	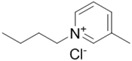		105		12		MCC Avicel		[33]		
triethyl-2-(2-methoxyethoxy)ethanammonium acetate ([Me(OEt)_3_-Et_3_N][OAc])	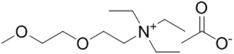		110		10		MCC Avicel		[32]		
1-octyl-3-methylimidazolium acetate ([Ocmim][OAc])	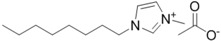		110		<1		Avicel		[32]		
1-(4,8,12-trioxatridecyl)-3-ethylimidazolium acetate ([Me(OPr)3-Et-Im][OAc])	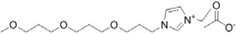		110		0.5		Avicel		[32]		

## 3. Chemical Modification of Cellulose in Ionic Liquids

**Figure 3:**
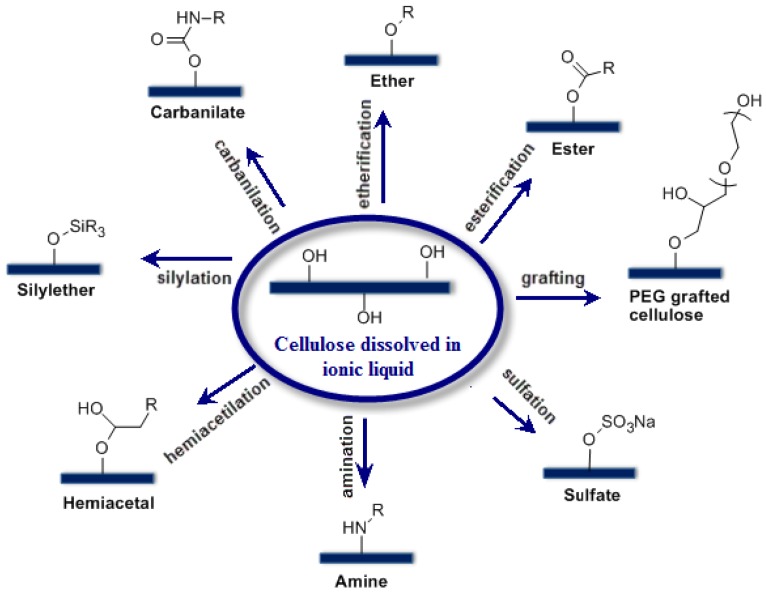
Possible chemical modifications that can be conducted to cellulose dissolved in ionic liquids.

While one direct application of cellulose dissolution in ionic liquids is their use as solvent media for the chemical modification of cellulose, some of the conventional solvent systems that are industrially used to process cellulose have been successfully utilized as a reaction media for homogeneous cellulose derivatizations [30,33,35,37,38,39,40,41]. However, the high toxicity, their tedious and only partial recovery have limited the use of conventional solvent systems for this purpose. Therefore, beginning from the introduction of the effective utilization of ionic liquids for the dissolution of cellulose, many studies have been carried out to use ionic liquids as reaction media for the functionalization of cellulose. A general reaction scheme for cellulose modifications is shown in Figure 3. Even though particular interest has been given to the acetylation of cellulose due to its many applications, other functionalizations to produce cellulose butyrate, cellulose phthalate, and cellulose benzoate have been carried out [42,43,44].

These functionalized cellulose derivatives find applications in different fields such as medicine, agriculture, or textile [45,46,47,48,49,50,51,52,53,54]. Aside from etherification and esterification reactions, cellulose derivatization with different functional groups such as silylates, sulfates, sulfonates, amines, hemiacetals, azides, carbanilates *etc.* [55,56,57,58,59] has been also successfully achieved by modifying the hydroxyl groups of cellulose. Finally, the work carried out by Tome *et al.* [60] where tetradecyltrihexylphosphonium bis(trifluoromethylsulfonyl)imide [TDTHP][NTF_2_] ionic liquid was used to solubilize cellulose and functionalize with various anhydrides should be mentioned. This particular ionic liquid can catalyze the esterification reaction. It was shown that the grafted fibers displayed higher surface hydrophobicity depending on the anhydride used for modification. Further characterization of the grafted fibers revealed that the esterification only took place at the fibers’ outmost layers indicating that the ultrastructure was not affected during the process.

Another interesting approach to modify cellulose is the grafting of polymer branches to cellulose to impart new properties without destroying its intrinsic characteristics [61]. Grafting to cellulose allows combining the properties of one or more polymers in one material. Hence, it is possible to acquire properties such as temperature responsiveness [62], hydrophobicity and oleophobicity [63], flexibility [63], sorbancy [62,64,65,66], ion exchange capability [65,67] and polarization [68]. These materials are promising candidates to replace the petroleum-based materials that are available in the market. Recently, ring-opening polymerization was used to modify cellulose. Generally, cyclic monomers such as epoxides, lactones, and anhydrides are used together with cellulose both dissolved in an appropriate ionic liquid. Hydroxyl groups of cellulose are then used to conduct ring-opening polymerization of the monomers to graft polymers to the cellulose backbone [69]. Another interesting work was conducted by Hufendiek *et al.* [70] where cellulose was transformed into a macro RAFT (Reversible Addition-Fragmentation chain Transfer) agent in 1-butyl-3-methylimidazolium chloride. This macro RAFT agent was used to conduct the grafting with polyacrylamide to result in temperature-responsive cellulose-graft-polyacrylamide copolymers. These copolymers were presented as new candidates for temperature responsive drug delivery systems. As it is seen from the examples given, ionic liquid dissolution of cellulose in mild conditions allows the conduction of homogeneous reactions that result in new materials with various properties. 

## 4. New Cellulosic Materials

### 4.1. Cellulose Composites

As discussed throughout the review, ionic liquids are ideal to dissolve cellulose, hence they have been extensively employed for the preparation of cellulose-polymer blends. The general process for the preparation of the blends is described next. First of all, cellulose and the polymer is dissolved together in the ionic liquid. Afterwards, the resulting homogeneous solution is precipitated to recover the blended polymeric mixture and to remove the ionic liquid. Along this line, Kadokawa *et al.* [71] used 1-butyl-3-methylimidazolium chloride as the solvent to blend cellulose with starch. Mixtures containing different cellulose-starch ratios were prepared and films were obtained by casting the homogeneous solution in between two glass plates followed by the removal of the solvent with acetone or ethanol. Similarly, polyamines were incorporated into cellulose with a similar preparation method in which cellulose solutions were mixed with different amounts of polyamines to form the corresponding composite materials [72]. The primary amines on the surface enabled the employment of these materials as solid support matrices for biocatalyst immobilization. This simple composite preparation process allows preparing composites as thin films or as beads that can be utilized for use in bioassays and supported reaction media. 

In another example, natural biopolymer wool was blended with cellulose by dissolving both materials in a mutual ionic liquid solvent such as 1-butyl-3-methylimidazolium chloride [73]. The solution containing both materials was precipitated in water giving a composite material with better thermal stability than the individual components. Moreover, mechanical strength of the composite was improved with increasing cellulose content and the elongation at break values were considerably enhanced with respect to individual components. It is also worth mentioning that the ionic liquid solvent was recycled with high yield and purity after composite preparation. 

### 4.2. Polymerized Ionic Liquid-Cellulose Composites

**Figure 4:**
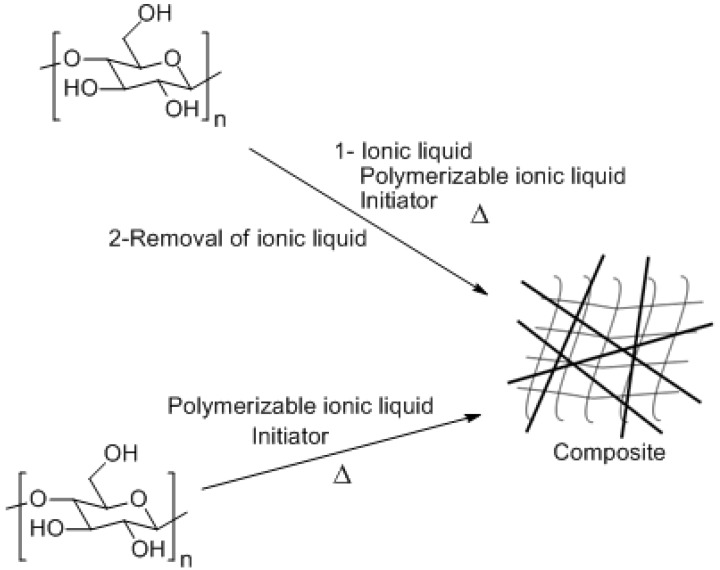
Two main approaches to obtain cellulose-poly (ionic liquid) composites. Δ = heat given to the system.

Aside from the utilization of ionic liquids as solvent, cellulose composites can also be prepared by direct polymerization of ionic liquid monomers in the presence of cellulose as depicted in Figure 4. Namely, one of the components is immobilized in the network of the other component [74,75,76]. As far as the cellulose solubility is concerned, imidazolium type ionic liquids have shown the best performance [16]. Therefore, imidazolium-based ionic liquid monomers are ideal candidates to combine with cellulose due to their high affinity and compatibility. For this reason, Murakami *et al.* [74] prepared imidazolium type ionic liquid monomer, 1-(3-acryloyloxybutyl)-3-methylimidazolium bromide (AcMIMBr). Eventually, the monomer and cellulose were dissolved in the ionic liquid 1-butyl-3-methylimidazolium chloride (BMIMCl) and the *in-situ* polymerization was carried out at elevated temperatures in the presence of an initiator. The composite material was purified by treating the final product with acetone followed by a methanol Soxhlet extraction. Differential scanning calorimetry (DSC) profile of the composite showed a sharp melting peak together with a clear glass transition temperature (T_g_) whereas polymerized ionic liquid displayed a single T_g_. The thermal degradation profile of the composite was similar to the polymerized ionic liquid homopolymer. Thermal findings of the composite material indicate that the cellulose was properly compatibilized with the polymerized ionic liquid system [74]. One drawback of this strategy can be asserted as the use of an ionic liquid solvent in addition to the use of an ionic liquid monomer, which requires the further cleaning of the final product to remove the free solvent. In order to overcome this issue, analogously, Kadokawa *et al.* [75] applied a slightly different approach to produce cellulose polymerized ionic liquid composites. In this particular case, the ionic liquid monomer contained a vinylbenzyl and vinyl functional groups that can produce an interpenetrating polymer network (IPN). First of all, cellulose was treated with the monomer for 24 h at 7 °C and afterwards it was polymerized at elevated temperature to produce the IPN. XRD (X-ray diffraction) analysis showed that in this case the crystalline structure of cellulose was only partially destroyed. Thermal properties of the composite materials were different than that of the individual components indicating good compatibility between the ionic liquid monomer and cellulose. 

Similarly, *in-situ* polymerization of ionic liquid monomers can also be utilized to produce cellulose based ionic porous materials. Hence, Prasad *et al.* [77] obtained a porous cellulose polymeric ionic liquid based material using templating technique with oil/ionic liquid emulsion system. In the first step, the cellulose was dissolved in BMIMCl and AcMIMBr, 1-(3-acryloyloxypropyl)-3-vinylimidazolium bromide (AcVIMBr) monomer mixture. After, the mixture was *in-situ* polymerized at elevated temperature. Then, the synthesized system was mixed with corn oil and sorbitan monooleate prior to the sonication process. The sonicated sample was treated with methanol, acetone, and hexane solvent system to produce the porous composite material. The composite displayed nonhomogeneous pore size distribution, pore sizes being in the range of 0.15–1.3 µm together with smaller sizes of 30–70 nm. 

Recently, our group developed a benign and facile *in* *situ* polymerization method to produce cellulose—polymerized ionic liquid composite coatings [78]. In this work, cellulose, treated with a methacrylic based quaternary ammonium ionic liquid monomer at room temperature, was applied onto a surface and photopolymerized in the presence of a photoinitiator. The resulting coating with 5 wt % of cellulose displayed good transparency indicating a good compatibility between the ionic liquid monomer and cellulose. 

### 4.3. Cellulose Based Ion Gels

**Figure 5:**
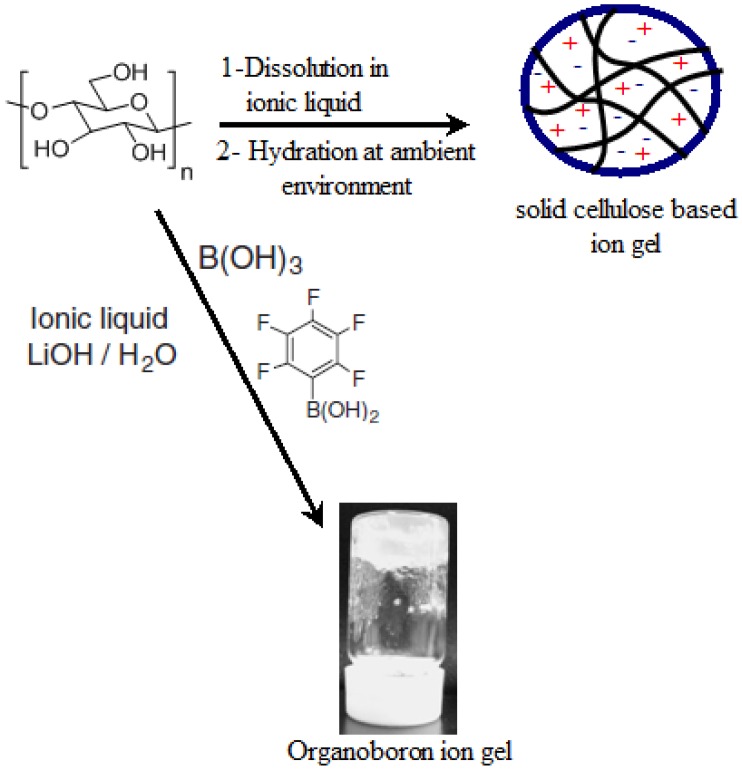
Two different approaches to produce cellulose containing ion gels.

As we mentioned before, 1-butyl-3-methylimidazolium chloride (BMIMCl) is one of the most effective ionic liquid solvents for breaking down the strong intra- and inter-molecular interactions that are present in cellulose. Interestingly, when cellulose is dissolved in this ionic liquid an unexpected gel formation could be observed after some days [79]. In the light of this information, the formation of the gel network was attributed to the development of noncrystalline cellulose aggregates in the solution upon the gradual absorption of water due to strong hygroscopic nature of BMIMCl. They found that when the gel was heated up to 120 °C, it became soft and gradually turned into a fluid at 150 °C. The soft material that was formed at 120 °C could be transformed into a gel by standing it at room temperature for 2 days. The regenerated gel displayed better transparency in spite of maintaining the flexibility due to more homogeneous distribution of the nano-domains with the heat treatment. Matsumi *et al.* [80] prepared ion gels that were produced through condensation reaction of cellulose with boric acid to give organoboron containing materials with improved conductivity. Cellulose was first dissolved in the ionic liquid and the condensation reaction was carried out in the solution with the addition of boric acid. The measured conductivities of the ion gels were comparable to the ionic liquid itself, meaning that the immobilization of the ionic liquid in the solid matrix did not induce a significant decrease in the conductivity. A general pathway to prepare cellulose based ion gels is displayed in Figure 5 below. Ion gels are a new class of materials that combine the properties of hydrogels (ionic character) and organogels [81]. They have many applications in the fields of electrolytic membranes, lithium-ion batteries, fuel cells, electrochemical- and bio-sensors, actuators and separation membranes [82,83,84,85,86,87]. Due to its technological and industrial interest, it is expected that the use of cellulose to generate ion gels will be more exploited in the near future and expanded to different types of ionic liquids.

### 4.4. Electrospinning of Cellulose from Ionic Liquids

**Figure 6:**
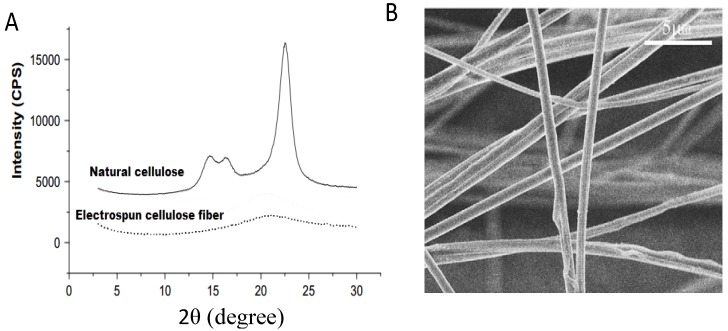
(**a**) XRD (X-ray diffraction) patterns of cellulose and electrospun fibers from AMIMCl; (**b**) SEM (scanning electron microscope) image of fibers produced from 5 wt % solution, reprinted from [89] with permission from Elsevier, Copyright 2008. CPS: count per second.

It is worth to remember that the industrial interest of dissolving cellulose from ionic liquids relies on the processing of cellulose fibers by classic spinning processes. Although the topic is out of the scope of this review is it worth to indicate it here and to indicate that it has been demonstrated already at the lab and semi-industrial level. On the other hand, other processes for producing fibers such as electrospinning of cellulose from conventional solvent systems was complicated due to their toxicity and harsh conditions required for dissolution. Hence, solubility of cellulose in ionic liquids facilitated the electrospinning process of this material to obtain fibers with tunable properties. Electrospun fibers of cellulose and cellulose derivatives find uses in the fields where other polymeric electrospun fibers are used such as filtration and biomedical applications [88]. One important aspect of electrospinning from ionic liquid systems is their nonvolatile nature; consequently they do not evaporate during the flight from the spinneret to the collector [89,90]. Therefore, the process requires a custom designed spinning procedure, in which the cellulose fibers are deposited into a coagulant media such as alcohol or water in order to recover the fibers and remove the ionic liquid. The fiber production process can be achieved through two main techniques; (a) dry-wet or (b) wet-wet electrospinning. In the first system, the solution is spun from the spinneret through an air-gap between nozzle and the coagulation bath. The formed fibers are immediately drawn out with a collector. In the latter system, the electrospun jet directly enters into the coagulation bath to form the fibers that are drawn out with a collector in the same way. The ionic liquid used to dissolve cellulose is removed from the fiber by passing through a coagulation bath in both two processes. For instance, Xu *et al.* [89] used a 1-allyl-3-methylimidazolium chloride (AMIMCl) and dimethylsulfoxide (DMSO) solvent system in order to produce electrospun cellulose fibers. The fibers were collected onto a rotating copper-wire drum under a high humidity and the remaining ionic liquid was eliminated resulting in a self-standing solid electrospun network (see Figure 6). XRD analysis revealed that the cellulose fibers were almost entirely amorphous after the electrospinning process.

Another study was performed by Ahn *et al.* [91] to investigate the effect of cosolvents on both electrospinnability and the resulting fiber morphology. 1-ethyl-3-methylimidazolium acetate was used as the solvent to dissolve cellulose and dimethylformamide (DMF) and dimethylacetamide (DMAc) were utilized as cosolvents. It was shown that the spinnability of cellulose increased as the concentration of cosolvent increases in the solution. Regardless of the cosolvent type, finer fibers were obtained with higher cosolvent concentrations, resulting in better web uniformity, thermal stability and crystallinity. DMF was found to be having more influence on fiber diameter and crystallinity. The reason behind the improvement of electrospinnability of cellulose with the addition of cosolvent is that the cosolvent penetrates into the gaps between the chains and the ionic liquid enhancing the chain mobility due to weaker interaction with the chains. As a result, cellulose chains can be easily elongated and fibrillated during the process. DMF allowed better elongation of the cellulose chains during the whipping process, which resulted in the formation of finer and more uniform fibers with higher crystallinity.

Besides using only cellulose, other polymeric materials can also be blended prior to the electrospinning process to create hybrid fibers with new properties. For instance, Viswanathan *et al.* prepared cellulose-heparin composites using ionic liquids [92]. They combined the blending process with electrospinning. Thus, two different solutions were prepared, on one hand cellulose was dissolved in BMIMCl and on the other hand heparin was dissolved in 1-ethyl-3-methylimidazolium benzoate. Prior to the electrospinning the two solutions were mixed and the blended fibers were collected in ethanol through precipitation of electrospun fibers. They found that the composite fibers displayed anticoagulant activity indicating that bioactivity of heparin remained unaffected. These composite fiber materials can be used for the construction of artificial blood vessels with excellent blood compatibility. Analogously, multiwall carbon nanotubes (MWCN) were successfully blended with cellulose using 1-ethyl-3-methylimidazolium chloride ionic liquid prior to the electrospinning process. Firstly, MWCN were dissolved in the ionic liquid to give a solid paste. Secondly, 1/5 of the desired amount of cellulose was dissolved in the ionic liquid at 80 °C and this solution was mixed with the previously prepared paste. Subsequently, remaining cellulose was added in small portions and mixed at 80 °C until a homogeneous mixture is obtained. Finally the mixture was spun into a coagulation bath containing ethanol as the coagulant (wet spinning) [93]. However, 0.05 mass fraction addition of carbon nanotubes resulted in 25% increase in the tensile strength and 100% increase in strain at break value without changing the modulus. The axial electrical conductivity at 0.1 mass fraction of carbon nanotubes was measured as 3000 S/m. These materials are promising candidates to be used for conductive textile fibers for electronic textile applications. 

## 5. Conclusions

Use of ionic liquids in new cellulose processing and materials technologies is a topic of burgeoning interest. The use of ionic liquids for cellulose dissolution stems from the unique properties of ionic liquids to interact with the strong hydrogen bonds of polysaccharides. The scientific discovery of the dissolution of cellulose in ionic liquids is being translated into new processing technologies, cellulose functionalization methods and new cellulose materials including blends, composites, fibers and ion gels. These materials can replace current analogs to overcome the environmental issues associated with petroleum-based products. Although there are many ionic liquids available that can dissolve cellulose, the processing difficulties such as fractionation need to be overcome to support large-scale use. However, due to the chemical versatility of both cellulose and ionic liquids new developments leading to the next generation of cellulosic materials are expected in the near future.
